# Chemical Constituents from the Roots of *Ranunculus ternatus* and their Inhibitory Effects on *Mycobacterium tuberculosis*

**DOI:** 10.3390/molecules181011859

**Published:** 2013-09-25

**Authors:** Ke-Zhong Deng, Ying Xiong, Bin Zhou, Yong-Mei Guan, Yong-Ming Luo

**Affiliations:** 1 Beijing University of Chinese Medicine, Beijing 100029, China; E-Mail: dengkezhong@126.com; 2 Jiangxi University of Traditional Chinese Medicine, Nanchang 330004, China; E-Mail: guanym2008@163.com; 3 Jiangxi Science and Technology Normal University, Nanchang 330038, China; E-Mail: tju_zhoubin@163.com

**Keywords:** *Ranunculus ternatus*, benzophenones, tuberculosis

## Abstract

Two new benzophenones, methyl (*R*)-3-[2-(3,4-dihydroxybenzoyl)-4,5-dihydroxyphenyl]-2-hydroxypropanoate (**1**) and *n*-butyl (*R*)-3-[2-(3,4-dihydroxybenzoyl)-4,5-dihydroxyphenyl]-2-hydroxypropanoate (**2**), were isolated from the roots of *Ranunculus ternatus* along with the two known compounds vanillic acid (**3**) and gallic acid (**4**). Their structures were elucidated by physical and spectroscopic analysis. In addition, compound **1** exhibited obvious activity against tuberculosis, while the activity of a 1:1 mixture of compound **1** plus **4** is better than that of **1** alone.

## 1. Introduction

*Ranunculus ternatus* Thunb. (Ranunculaceae) is mainly distributed in the Henan and Anhui region of China and has been used in traditional Chinese medicine for the treatment of tuberculosis, faucitis and neck scrofula. Several lactones [1,2], flavonoids [3,4], triterpenoids [5], glycosides [6] and two alkaloids [7] have been isolated from the roots of this plant. Our previous phytochemical investigations on the crude drug revealed benzophenones [8]. It was reported that the extracts of Radix Ranuncoli Ternati such as the organic acid fraction had antimycobacterial activity *in vitro* [9]. However, very few reports have looked into the active ingredient(s) with antituberculosis effects, apart from ternatolide [10]. Here we describe the isolation of two novel benzophenones (compounds **1**, **2**) and two known substituted benzene compounds (compounds **3**, **4**) from *R*. *ternatus*, structure elucidation on the novel compounds and their inhibitory activity against *M. tuberculosis*. 

## 2. Results and Discussion

**Figure 1:**
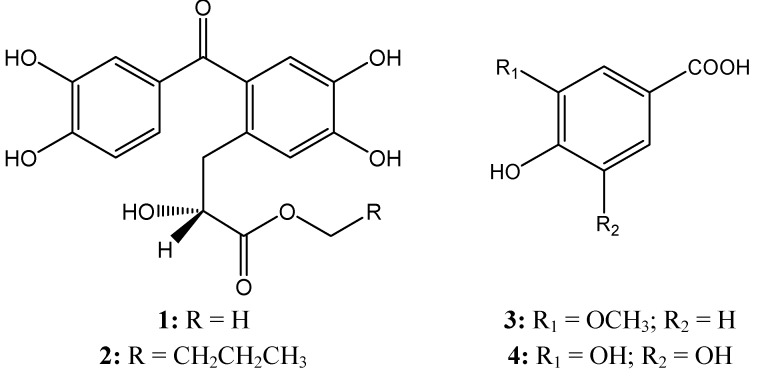
The structures of compounds **1**–**4**.

The ethanol extract of the roots of *R*. *ternatus* was partitioned using various solvents (see Section 3) and the H_2_O soluble fraction was subsequently purified by repeated column chromatography (macroporous resin, ODS and Sephadex LH-20) to afford compounds **1**–**4** (Figure 1).

**Figure 2:**
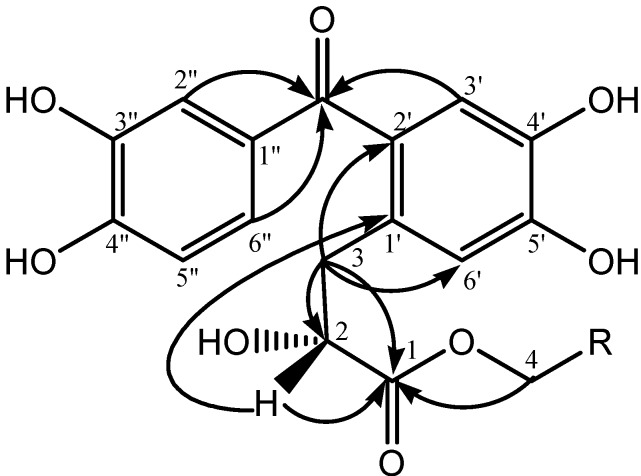
Structures and key HMBC (H→C) correlations of compounds **1** and **2**.

Compound **1** was obtained as a yellowish gum, 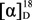 +30.2 (c 0.1, MeOH). Its molecular formula was established to be C_17_H_16_O_8_ by HRESI-MS (*m/z* 349.0919, calcd. 349.0918 [M+H]^+^). The IR bands at 3,264, 1,730, 1,590 and 1,519 cm^−1^ revealed the presence of hydroxyl, carbonyl and aromatic ring groups. The ^1^H-NMR spectrum (Table 1) of compound **1** show three aromatic protons signals at *δ*_H_ 7.28 (d, 1H, *J* = 2.0 Hz), 7.17 (dd, 1H, *J* = 8.0 and 2.0 Hz), 6.82 (d, 1H, *J* = 8.0 Hz) as an ABX-type system and two at *δ*_H_ 6.79 (s, 2H). In addition, one methylene at *δ*_H_ 3.04 (dd, 1H, *J* = 13.6 and 5.2 Hz), 2.91 (dd, 1H, *J* = 13.6 and 8.0 Hz) and one oxygenated methine at *δ*_H_ 4.30 (dd, 1H, *J* = 8.0 and 5.2 Hz), as well as one methoxyl group at *δ*_H_ 3.64 (s, 3H) were observed. The ^13^C-NMR spectrum (Table 1) combined with HSQC of compound **1** exhibited the signals for 17 carbons, including two phenyls, one carbonyl ketone and one carbonyl ester groups *etc*. In the HMBC spectrum (Figure 2), the correlation between H-3', H-2'', H-6'' and C=O (*δ*_C_ 197.7) displayed that compound **1** possessed a diphenylketone skeleton. Moreover, H-3 correlated with C-1, C-2, C-2' and C-6'; H-2 correlated with C-1 and C-1'; H-4 correlated with C-1. The absolute configuration of C-2 was determined as R by comparing its CD spectrum [219 (∆ε −7.38), 289 nm (∆ε −0.618)] with that of ethyl (*S*)-3-[2-(3,4-dihydroxybenzoyl)-4,5-dihydroxyphenyl]-2-hydroxypropanoate [219 (∆ε +15.5), 273 nm (∆ε +1.99)] isolated from *R*. *ternatus* [8,11,12]. Based on the above evidence, the structure of compound **1** was elucidated to be methyl (*R*)-3-[2-(3,4-dihydroxybenzoyl)-4,5-dihydroxyphenyl]-2-hydroxypropanoate. 

**Table 1:** ^1^H (400 MHz) and ^13^C (100 MHz) NMR data of compounds **1** and **2** (in CD_3_OD, δ ppm, *J* Hz).

No.	1		2	
	δ_H_	δ_C_	δ_H_	δ_C_
1		174.4		174.2
2	4.30 (dd, 1H, 8.0, 5.2)	71.9	4.25 (dd, 1H, 8.0, 5.6)	71.9
3	3.04 (dd, 1H, 13.6, 5.2)	36.8	3.00 (dd, 1H, 13.6, 5.6)	37.0
	2.91 (dd, 1H, 13.6, 8.0)		2.93 (dd, 1H, 13.6, 8.0)	
4	3.64 (s, 3H)	50.9	4.02 (t, 2H, 6.8)	64.3
5			1.52 (m, 2H)	30.3
6			1.29 (m, 2H)	18.6
7			0.90 (t, 3H, 7.2)	12.6
CO		197.7		197.7
1'		128.9		128.7
2'		130.3		130.4
3'	6.79 (s, 1H)	117.1	6.80 (s, 1H)	117.0
4'		142.6		142.7
5'		147.5		147.4
6'	6.79 (s, 1H)	117.9	6.80 (s, 1H)	118.0
1''		130.1		130.1
2''	7.28 (d, 1H, 2.0)	116.8	7.29 (d, 1H, 2.0)	116.8
3''		144.8		144.9
4''		150.9		150.9
5''	6.82 (d, 1H, 8.0)	114.2	6.81 (d, 1H, 8.0)	114.2
6''	7.17 (dd, 1H, 8.0, 2.0)	124.4	7.17 (dd, 1H, 8.0, 2.0)	124.4

**1:** R = H; **2:** R = CH_2_CH_2_CH_3_.

Compound **2** was obtained as a yellowish gum, 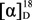 +19.0 (c 0.06, MeOH). Its HRESI-MS showed [M+H]^+^ at *m/z* 391.1385 (calcd. 391.1387), corresponding to the molecular formula C_20_H_22_O_8_. Its IR spectrum indicated the presence of hydroxyl (3,291 cm^−1^), carbonyl (1,728 cm^−1^) and aromatic rings (1,592 and 1,519 cm^−1^). In comparison with compound **1**, the spectra (^1^H-, ^13^C-NMR, ^1^H-^1^H COSY, HSQC and HMBC) are quite similar, except for the methoxyl group of compound **1**, which was replaced by an *n*-butoxy group (*δ*_c_ 64.3, 30.3, 18.6, 12.6) of compound **2**. By analysis of the HMBC spectrum, the correlation of H-4 correlated with C-1 indicated that the *n*-butoxy moiety was linked at the C-1 position. Moreover, the CD spectrum showed a negative Cotton effect at 219 (*∆ε* −7.09) and 276 nm (*∆ε* −0.502) [8,9], indicating the R-configuration of C-2. Thus, compound **2** was elucidated to be *n*-butyl (*R*)-3-[2-(3,4-dihydroxybenzoyl)-4,5-dihydroxyphenyl]-2-hydroxypropanoate.

The two known compounds, vanillic acid (**3**) and gallic acid (**4**), were identified by comparison of their physical and spectroscopic data with literature values [13]. All the compounds were evaluated for *in vitro* anti-tuberculosis activity against *M*. *tuberculosis* H_37_Rv (Table 2). Compounds **1**, **2**, vanillic acid (**3**) and gallic acid (**4**) are responsible for the antimycobacterial activity observed in *R*. *ternatus*.Compound **1** (MIC = 41.67 ± 14.43 µg/mL) was the most active one. Furthermore, the activity of a 1:1 combination of compounds **1** and **4** (MIC = 20.83 ± 7.22 µg/mL) was better than that of **1** alone. 

**Table 2:** MIC values (µg/mL) of constituents from *R*. *ternatus* against *M*. *tuberculosis*.

Sample						Control
1	2	3	4	Mixture of 1 + 3 (1:1)	Mixture of 1 + 4 (1:1)	
41.67 ± 14.43 *	266.67 ± 115.47	83.33 ± 28.87 *	66.67 ± 28.87	83.33 ± 28.87 *	20.83 ± 7.22 *	2.08 ± 0.90

Each value represents the mean ± S.D. (*n* = 3). Compared with sample of control group. * *p* < 0.05.

## 3. Experimental

### 3.1. General Procedures

Optical rotations were measured on a WZZ-1 automatic polarimeter. IR spectra were obtained on a Bio-Rad FTS 6000 infrared spectrometer. HRESIMS spectra were performed on an Ionspec 7.0 T FTICR MS. 1D- and 2D-NMR spectra were recorded on a Bruker AVANCE-400 (400 MHz for ^1^H- and 100 MHz for ^13^C-) NMR spectrometer using TMS as an internal standard. Preparative HPLC was carried out on an ODS column (250 × 20 mm, YMC) with a CXTH LC-3000 UV-detector. Silica gel (200–300 mesh, Qingdao Ocean Chemical Group Co., Qingdao, China) and Sephadex LH-20 (Merck Co., Darmstadt, Germany) for column chromatography as well as silica gel GF254 (Qingdao Ocean Chemical Group Co. of China) for TLC were used.

### 3.2. Plant Material

The roots of *Ranunculus ternatus* were purchased from Zhangshu, Jiangxi Province, China and was identified by Associate Prof. Ke-Zhong Deng. A voucher specimen (voucher specimen No. RT1101) is deposited in the School of Pharmacy, Jiangxi University of Traditional Chinese Medicine, China. 

### 3.3. Extraction and Isolation

The roots of *Ranunculus ternatus* (25 kg) were powdered and successively extracted with 95% and 65% EtOH (100 L, 2 h × 3, respectively) under reflux for two hours and then filtered. After removal of the solvent under reduced pressure, the residue (4 kg) was suspended in water and partitioned with light petroleum ether and EtOAc successively. The remaining water soluble fraction (2,580 g) was passed through a macroporous resin column (Amberlite XAD 16) and eluted with H_2_O-EtOH (1:0→0:95, *v/v*) to give 12 fractions. Fraction 5 (12 g) was loaded on an open ODS column and eluted with H_2_O-MeOH (9:1→0:1, *v/v*) to give nine subfractions. Sub-fraction 4 was subjected to preparative HPLC (YMC-pack ODS-A, 250 mm × 20 mm, MeOH/H_2_O 3:7, 5 mL/min) to afford compound **1** (24 mg) and **4** (18 mg), Sub-fraction 6 was separated by preparative HPLC (YMC-pack ODS-A, 250 mm × 20 mm, MeOH/H_2_O 5:5, 5 mL/min) and purified on a Sephadex LH-20 column eluted with MeOH to yield compounds **2** (38 mg) and **3** (8 mg). 

#### 3.3.1. Methyl (R)-3-[2-(3,4-dihydroxybenzoyl)-4,5-dihydroxyphenyl]-2-hydroxypropanoate (**1**)

Yellowish gum; 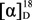 +30.2 (c 0.10, MeOH); IR bands (KBr): 3,264, 2,957, 1,731, 1,591, 1,519, 1,441, 1,366, 1,296, 1,222, 1,160, 1,115, 1,024, 889, 833, 783, 771, 631 cm^−1^; positive ion HRESIMS *m/z*: [M+H]^+^ 349.0919 for C_17_H_16_O_8_ + H (calcd. 349.0918); ^1^H-NMR (CD_3_OD, 400 MHz) and ^13^C-NMR (CD_3_OD, 100 MHz) are shown in Table 1.

#### 3.3.2. n-Butyl (R)-3-[2-(3,4-dihydroxybenzoyl)-4,5-dihydroxyphenyl]-2-hydroxypropanoate "(**2**)

Yellowish gum; 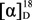 +19.0 (c 0.06, MeOH); IR bands (KBr): 3,291, 2,961, 1,728, 1,592, 1,518, 1,441, 1,372, 1,295, 1,216, 1,160, 1,113, 1,083, 892, 834, 783, 630 cm^−1^; positive ion HRESIMS *m/z*: [M+H]^+^ 391.1385 for C_17_H_16_O_8_ + H (calcd. 391.1387); ^1^H-NMR (CD_3_OD, 400 MHz) and ^13^C-NMR (CD_3_OD, 100 MHz) are shown in Table 1.

### 3.4. Anti-Tuberculosis Activity

Anti-mycobacterial activities of compounds **1**–**4** against *M*. *tuberculosis* strains H_37_Rv (strains were obtained from the Jiangxi Province Centers for Disease Prevention and Control, Nanchang, China) were evaluated by the Microplate Alamar Blue Assay [14]. The MIC (minimum inhibitory concentration) values were determined and compared with rifampicin as a reference drug.

## 4. Conclusions

Two new benzophenones (compounds **1**, **2**) together with two known organic acids (compounds **3**, **4**) were isolated from *R*. *ternatus* Thunb. Compounds **1**–**4** were assayed for their anti-tuberculosis activity and the data proved that comound **1** had significant inhibitory activity, furthermore, the activity of a mixture of compounds **1** and **4** is better than that of **1** alone. Up to now, natural benzophenones that exhibited anaphylaxis, inflammatory, alpha-glucosidase and HIV inhibition activity have been reported from species of the Clusiaceae, Thymelaeaceae, Myrtaceae and Iridaceae, *etc.* [15,16,17,18,19]. The substituents on the phenyl ring usually are hydroxyl, methoxy and isopentene groups. Benzophenones which possess propionate ester groups with a chiral carbon were only found in *R*. *ternatus* of the Ranunculaceae. This is the first report about benzophenones as potential anti-tuberculosis inhibitors.

## Supplementary Materials

Supplementary materials can be accessed at: http://www.mdpi.com/1420-3049/18/10/11859.
