# Supplementary material for: Chemical Constituents from the Roots of Ranunculus ternatus and their Inhibitory Effects on Mycobacterium tuberculosis
Source: Molecules. 2013 Sep 25;18(10):11859–65. doi: 10.3390/molecules181011859 (PMC6270127; doi:10.3390/molecules181011859)
Supplement: Supplementary file 1 [file molecules-18-11859-s001.doc]

Supplementary Materials

**Figure S1.** Main HMBC Correlations of Compounds **1** and **2**.

**1**: R = H; **2**: R = CH_2_CH_2_CH_3_.

**Figure S2.** The ^1^H-NMR Spectrum of Compound **1** in CD_3_OD.

**Figure S3.** The ^13^C-NMR Spectrum of Compound **1** in CD_3_OD.

**Figure S4.** The HSQC Spectrum of Compound **1** in CD_3_OD.

**Figure S5.** The HMBC Spectrum of Compound **1** in CD_3_OD.

**Figure S6.** The ^1^H-^1^H COSY Spectrum of Compound **1** in CD_3_OD.

**Figure S7.** The IR Spectrum of Compound **1**.

**Figure S8.** The HR-MS Spectrum of Compound **1**.

**Figure S9.** The ^1^H-NMR Spectrum of Compound **2** in CD_3_OD.

**Figure S10.** The ^13^C-NMR Spectrum of Compound **2** in CD_3_OD.

**Figure S11.** The HSQC Spectrum of Compound **2** in CD_3_OD.

**Figure S12.** The HMBC Spectrum of Compound **2** in CD_3_OD.

**Figure S13.** The ^1^H-^1^H COSY Spectrum of Compound **2** in CD_3_OD.

**Figure S14.** The IR Spectrum of Compound **2**.

**Figure S15.** The HR-MS Spectrum of Compound **2**.
